# Effect of a New Prokinetic Agent DA-9701 Formulated with Corydalis Tuber and Pharbitidis Semen on Cytochrome P450 and UDP-Glucuronosyltransferase Enzyme Activities in Human Liver Microsomes

**DOI:** 10.1155/2012/650718

**Published:** 2012-04-02

**Authors:** Hye Young Ji, Kwang Hyeon Liu, Ji Hyeon Jeong, Dae-Young Lee, Hyun Joo Shim, Miwon Son, Hye Suk Lee

**Affiliations:** ^1^Drug Metabolism and Bioanalysis Laboratory, College of Pharmacy, The Catholic University of Korea, 43-1 Yeokgok 2-dong, Wonmi-gu, Gyeonggi-do, Bucheon, 420-743, Republic of Korea; ^2^College of Pharmacy and Research Institute of Pharmaceutical Sciences, Kyungpook National University, Daegu 702-701, Republic of Korea; ^3^Research Center, Dong-A Pharmaceutical Co., Yongin 446-905, Republic of Korea

## Abstract

DA-9701 is a new botanical drug composed of the extracts of Corydalis tuber and Pharbitidis semen, and it is used as an oral therapy for the treatment of functional dyspepsia in Korea. The inhibitory potentials of DA-9701 and its component herbs, Corydalis tuber and Pharbitidis semen, on the activities of seven major human cytochrome P450 (CYP) enzymes and four UDP-glucuronosyltransferase (UGT) enzymes in human liver microsomes were investigated using liquid chromatography-tandem mass spectrometry. DA-9701 and Corydalis tuber extract slightly inhibited UGT1A1-mediated etoposide glucuronidation, with 50% inhibitory concentration (IC_50_) values of 188 and 290 *μ*g/mL, respectively. DA-9701 inhibited CYP2D6-catalyzed bufuralol 1′-hydroxylation with an inhibition constant (*K*_i_) value of 6.3 *μ*g/mL in a noncompetitive manner. Corydalis tuber extract competitively inhibited CYP2D6-mediated bufuralol 1′-hydroxylation, with a *K*_i_ value of 3.7 *μ*g/mL, whereas Pharbitidis semen extract showed no inhibition. The volume in which the dose could be diluted to generate an IC_50_ equivalent concentration (volume per dose index) value of DA-9701 for inhibition of CYP2D6 activity was 1.16 L/dose, indicating that DA-9701 may not be a potent CYP2D6 inhibitor. Further clinical studies are warranted to evaluate the *in vivo* extent of the observed *in vitro* interactions.

## 1. Introduction

Functional dyspepsia is a very common chronic gastrointestinal disorder, with a prevalence of 40% using the more liberal criteria [1]. Although the etiology and pathogenesis of functional dyspepsia are poorly understood, pathophysiologic abnormalities such as delayed gastric emptying and impaired gastric accommodation have been reported in 30–40% of functional dyspepsia patients [2–5]. Medications used for the treatment of functional dyspepsia have been developed based on the pathophysiological mechanisms associated with functional dyspepsia; those medications include prokinetic agents, which are now restricted due to serious adverse effects and failure to confirm their efficacy. Therefore, it is necessary to develop safer and more effective drugs for the treatment of functional dyspepsia.

DA-9701 is a new botanical drug composed of the extracts of Corydalis tuber from the roots of *Corydalis yanhusuo* W. T. Wang and Pharbitidis semen from the seed of *Pharbitis nil* Choisy, and it is used as an oral therapy (Motilitone^®^) for the treatment of functional dyspepsia (FD) in Republic of Korea since May 2011 [6–8]. Pharbitidis semen has been used as a folk medicine for its analgesic effect on abdominal disorders. Corydalis tubers from the roots of *Corydalis yanhusuo* W. T. Wang have long been used as a herbal drug for their analgesic and antiulcer effects [9–11]. Corydaline and tetrahydroberberine, isoquinoline alkaloids of Corydalis tubers, promote gastric emptying and facilitate gastric accommodation [12, 13]. DA-9701 has been demonstrated to show strong gastroprokinetic effects and a safety profile superior to cisapride and mosapride [7, 8]. The gastroprokinetic effects of DA-9701 might be mediated by the induction of pacemaker currents in the interstitial cells of Cajal (ICC) [6]. DA-9701 also has been shown to have antagonistic effects on the D2 receptor and agonistic effects on 5-HT4, 5-HT1a, and 5-HT1b receptors [7, 8].

Since botanical drugs share the same drug metabolizing enzymes, including cytochrome P450 (CYP) enzymes, UDP-glucuronosyltransferase (UGT) enzymes, and drug transporters, such as multidrug resistance protein and p-glycoprotein, with commonly used drugs, the potential for herb-drug interaction is substantial [14–16]. Several medicinal herbs, including Dong quai (*Angelica polymorpha*), ginkgo (*Ginkgo biloba*), ginseng (*Panax ginseng*), milk thistle (*Silybum marianum*), licorice (*Glycyrrhiza glabra*), St. John's wort (*Hypericum perforatum*), and Woohwangcheongsimwon have been reported to cause herb-drug interactions [17–20]. Herb-drug interactions have been identified with St. John's wort that significantly involve reduced blood concentrations of cyclosporine, digoxin, midazolam, indinavir, tacrolimus, theophylline, and warfarin [21]. Undoubtedly, the early identification of herb-drug interactions is imperative to prevent potentially dangerous clinical outcomes.

To the best of our knowledge, no previous study has reported the effects of Corydalis tubers and Pharbitidis semen, the component herbs of DA-9701, on human CYP and UGT enzymes. In this study, the effects of DA-9701 and its component herbs, Corydalis tubers and Pharbitidis semen, on the activities of seven major human CYP enzymes and four major human UGTs, 1A1, 1A4, 1A9, and 2B7, were examined using pooled human liver microsomes in order to evaluate the possibility of drug interactions of DA-9701.

## 2. Experimental

### 2.1. Materials and Reagents

Corydaline, palmatine, and chlorogenic acid were obtained from Wako Pure Chemical Industries, Ltd. (Osaka, Japan). Acetaminophen, alamethicin, coumarin, diclofenac, etoposide, glucose-6-phosphate, glucose-6-phosphate dehydrogenase, 7-hydroxycoumarin, midazolam, *β*-nicotinamide adenine dinucleotide phosphate reduced form (NADPH), phenacetin, propofol, trifluoperazine, and uridine-5-diphosphoglucuronic acid trisodium salt (UDPGA) were purchased from Sigma Chemical Co. (St. Louis, MO, USA). Pooled human liver microsomes (H161), ^13^C_2_, ^15^N-acetaminophen, bufuralol, *N*-desethylamodiaquine, 1′-hydroxybufuralol, d_9_-1′-hydroxybufuralol maleate, 4-hydroxydiclofenac, ^13^C_6_-4-hydroxydiclofenac, 4-hydroxymephenytoin, d_3_-4-hydroxy-mephenytoin, 1′-hydroxymidazolam, and (*S*)-mephenytoin were obtained from BD Gentest Co. (Woburn, MA, USA). Azidothymidine, azidothymidine glucuronide, and propofol glucuronide were obtained from Toronto Research Center (Toronto, Canada). Acetonitrile and methanol (HPLC grade) were obtained from Burdick & Jackson Inc. (Muskegon, MI, USA), and the other chemicals were of the highest quality available.

### 2.2. Preparation of DA-9701

DA-9701 is a standardized extract of Corydalis tuber and Pharbitidis semen which was prepared as previously reported [8]. Briefly, those dried herbs were mixed in a specific ratio (5 : 1) and extracted with 50% aqueous ethanol three times at room temperature for 48 h. After filtration, the aqueous ethanol extract was evaporated under reduced pressure and lyophilized for a complete removal of the residual solvent to yield brown powder. Contents of two marker components, corydaline, an alkaloid, from Corydalis tuber and chlorogenic acid from Pharbitidis semen in DA-9701, were determined by high performance liquid chromatography (HPLC) [8].

Corydalis tuber extract and Pharbitidis semen extract were prepared as previously described [13]. Briefly, dried Corydalis tuber and Pharbitidis semen were separately extracted with 50% aqueous ethanol three times at room temperature for 48 h. The solvents were removed under vacuum. The marker compounds including corydaline from Corydalis tuber extract and chlorogenic acid from Pharbitidis semen extract were also determined by HPLC [8]. The two raw materials were purchased in China.

### 2.3. Inhibitory Effects of DA-9701 and Its Component Herbs on Seven Major CYP Activities in Human Liver Microsomes

Inhibitory potencies (IC_50_ values) of DA-9701, Corydalis tuber extract, and Pharbitidis semen extract were determined using CYP assays in the presence and absence of DA-9701, Corydalis tuber extract, and Pharbitidis semen extract using pooled human liver microsomes (H161, Gentest). Phenacetin *O*-deethylase, coumarin 7-hydroxylase, amodiaquine *N*-deethylase, diclofenac 4-hydroxylase, (*S*)-mephenytoin 4-hydroxylase, bufuralol 1′-hydroxylase, and midazolam 1′-hydroxylase activities were determined as probe activities for CYP1A2, CYP2A6, CYP2C8, CYP2C9, CYP2C19, CYP2D6, and CYP3A, respectively, using cocktail incubation and liquid chromatography-tandem mass spectrometry (LC/MS/MS) [22]. The incubation mixtures were prepared in a total volume of 200 *μ*L as follows: pooled human liver microsomes (0.25 mg/mL), 3.3 mM MgCl_2_, 50 mM potassium phosphate buffer (pH 7.4), and a cocktail of probe substrates and various concentrations of DA-9701, Corydalis tuber extract, or Pharbitidis semen extract (final concentrations of 1–200 *μ*g/mL with an acetonitrile concentration less than 0.5% v/v). The substrates were used at concentrations approximately equal to their respective *K*_*m*_ values: 50 *μ*M phenacetin, 2.5 *μ*M coumarin, 2.5 *μ*M amodiaquine, 10 *μ*M diclofenac, 100 *μ*M (*S*)-mephenytoin, 5 *μ*M bufuralol, and 2.5 *μ*M midazolam. After a 3-min preincubation period at 37°C, the reactions were initiated by addition of an NADPH (final concentration of 1.3 mM) generating system and incubated for 20 min at 37°C in a shaking water bath. After incubation, the reaction was stopped by placement of the tubes on ice and addition of 100 *μ*L of ice-cold methanol containing internal standards (^13^C_2_, ^15^N-acetaminophen for acetaminophen and *N*-desethylamodiaquine, d_5_-7-hydroxycoumarin for 7-hydroxycoumarin, ^13^C_6_-4-hydroxydiclofenac for 4-hydroxydiclofenac, d_3_-4-hydroxy-mephenytoin for 4-hydroxymephenytoin and 1′-hydroxymidazolam, d_9_-1′-hydroxybufuralol for 1′-hydroxybufuralol). The incubation mixtures were then centrifuged at 13,000 × g for 5 min. All incubations were performed in triplicate, and the mean values were used. For evaluation of NADPH-dependent mechanism-based inhibition of CYP activities, various concentrations of DA-9701, Corydalis tuber extract, or Pharbitidis semen extract (1–200 *μ*g/mL) were pre-incubated for 30 min with human liver microsomes in the presence of NADPH. The reaction was started by addition of a cocktail of CYP probe substrates.

All seven metabolites produced from the cocktail incubation of CYP isoform-specific substrates were simultaneously determined by LC/MS/MS [22]. The system consisted of a tandem mass spectrometer (TSQ Quantum Access, ThermoFisher Scientific, San Jose, CA, USA) coupled with a Nanospace SI-2 LC system (Tokyo, Japan). Separation was performed on an Atlantis dC18 column (5 *μ*m, 2.1 mm i.d. ×100 mm, Waters, MA, USA) using the gradient elution of a mixture of 5% methanol in 0.1% formic acid (mobile phase A) and 95% methanol in 0.1% formic acid (mobile phase B) at a flow rate of 0.25 mL/min: 10% mobile phase B for 1 min, 10% to 95% mobile phase B for 1 min, and 95% mobile phase B for 5 min. The column and autosampler temperatures were 50 and 6°C, respectively. After 1.5 min, the LC eluent was diverted from waste to the mass spectrometer fitted with the electrospray ionization (ESI) source and operated in positive ion mode. ESI source settings for ionization of the metabolites were as follows: electrospray voltage, 5.0 kV; vaporizer temperature, 420°C; capillary temperature 360°C; sheath gas pressure, 35 psi; auxiliary gas pressure, 10 psi. Quantification was performed by selected reaction monitoring (SRM) of the [M + H]^+^ ion and the related product ion for each metabolite. SRM transitions for the metabolites and internal standards are summarized in our previous paper [22]. Analytical data were processed using Xcalibur software (Thermo Fisher Scientific).

### 2.4. Inhibitory Effects of DA-9701 and Its Component Herbs on Four UGT Activities in Human Liver Microsomes

The inhibitory potencies (IC_50_ values) of DA-9701, Corydalis tuber extract, or Pharbitidis semen extract were also determined with UGT assays in the presence and absence of DA-9701, Corydalis tuber extract, or Pharbitidis semen extract using pooled human liver microsomes. Etoposide glucuronidation, trifluoperazine glucuronidation, propofol glucuronidation, and azidothymidine glucuronidation activities were determined as probe activities for UGT1A1, UGT1A4, UGT1A9, and UGT2B7, respectively, using LC/MS/MS [23]. Incubation mixtures were prepared in a total volume of 100 *μ*L as follows: pooled human liver microsomes (0.2 mg/mL for etoposide, trifluoperazine, and azidothymidine; 0.1 mg/mL for propofol), 25 *μ*g/mL alamethicin, 10 mM MgCl_2_, 50 mM tris buffer (pH 7.4), each UGT-isoform specific probe substrate (200 *μ*M etoposide for UGT1A1, 5 *μ*M trifluoperazine for UGT1A4, 10 *μ*M propofol for UGT1A9, or 100 *μ*M azidothymidine for UGT2B7), and various concentrations of DA-9701, Corydalis tuber extract, or Pharbitidis semen extract (final concentrations of 1–200 *μ*g/mL with acetonitrile concentration less than 0.5% v/v). Reactions were initiated by addition of UDPGA (final concentration of 5 mM), and incubations were carried out at 37°C in a shaking water bath for 30 min. Reactions were terminated by addition of 100 *μ*L of ice-cold methanol containing an internal standard (100 ng/mL ezetimibe for etoposide glucuronide and propofol glucuronide; 30 ng/mL meloxicam for trifluoperazine glucuronide and azidothymidine glucuronide). The incubation mixtures were centrifuged at 13,000 × g for 5 min, followed by dilution of 40 *μ*L of the supernatant with 60 *μ*L of water. The aliquot (5 *μ*L) was injected onto LC/MS/MS. All incubations were performed in duplicate and the mean values were used.

Glucuronides produced from UGT isoform-specific substrates were, respectively, determined by LC/MS/MS [23]. Separation was performed on an Atlantis dC18 column (5 *μ*m, 2.1 mm i.d. ×100 mm, Waters, MA, USA) using the gradient elution of a mixture of 5% methanol in 0.1% formic acid (mobile phase A) and 95% methanol in 0.1% formic acid (mobile phase B) at a flow rate of 0.25 mL/min: 10% mobile phase B for 2 min, 10% to 95% mobile phase B for 4 min. The column and autosampler temperatures were 50 and 6°C, respectively. After 3.0 min, the LC eluent was diverted from waste to the mass spectrometer fitted with an ESI source. ESI source settings for ionization of trifluoperazine glucuronide and azidothymidine glucuronide in positive ion mode were as follows: electrospray voltage, 5.0 kV; vaporizer temperature, 420°C; capillary temperature 360°C; sheath gas pressure, 35 psi; auxiliary gas pressure, 10 psi. ESI source settings for ionization of etoposide glucuronide and propofol glucuronide in negative ion mode were as follows: electrospray voltage, −4.0 kV; vaporizer temperature, 420°C; capillary temperature 360°C; sheath gas pressure, 35 psi; auxiliary gas pressure, 10 psi. Quantification was performed by SRM of the [M + H]^+^ ion for trifluoperazine glucuronide and azidothymidine glucuronide or [M − H]^−^ for etoposide glucuronide and propofol glucuronide and the related product ion for each metabolite. SRM transitions for the metabolites and internal standard are summarized in our previous paper [23]. Analytical data were processed using Xcalibur software (Thermo Fisher Scientific).

### 2.5. Kinetic Analysis

In order to determine *K*_i_ values of DA-9701, Corydalis tuber extract, and a typical inhibitor quinidine for CYP2D6 enzyme, human liver microsomes (0.1 mg/mL) were incubated with various concentrations of 0.5 − 5 *μ*M bufuralol, 10 mM MgCl_2_, and various concentrations of DA-9701, Corydalis tuber extract, or quinidine in 50 mM potassium phosphate buffer (pH 7.4) in a total incubation volume of 200 *μ*L. Reactions were initiated by addition of NADPH (final concentration of 1 mM) at 37°C and stopped after 15 min by placement of the incubation tubes on ice and addition of 100 *μ*L of ice-cold methanol containing an internal standard (0.5 *μ*g/mL d_9_-1′-hydroxybufuralol). The incubation mixtures were centrifuged at 13,000 × g for 5 min, and aliquots (5 *μ*L) of the supernatants were analyzed by LC/MS/MS.

### 2.6. Data Analysis

IC_50_ values (concentration of inhibitor causing 50% inhibition of the original enzyme activity) were calculated using WinNonlin software, a nonlinear regression analysis program (Pharsight, Mountain View, CA, USA). Apparent kinetic parameters for inhibitory potential (*K*_i_ values) were estimated from the fitted curves using Enzyme Kinetics Ver. 1.3 program (Systat Software Inc., San Jose, CA, USA).

## 3. Results and Discussion

The inhibitory effects of DA-9701, Corydalis tuber extract, and Pharbitidis semen extract on seven major human CYP isoforms were evaluated using cocktail CYP probe substrates in human liver microsomes. CYP2D6-catalyzed bufuralol 1′-hydroxylation activity was inhibited by DA-9701 and Corydalis tuber extract with IC_50_ values of 25.9 and 15.8 *μ*g/mL, respectively, but not by Pharbitidis semen extract (Table 1). DA-9701, Corydalis tuber extract, and Pharbitidis semen extract at 200 *μ*g/mL showed negligible inhibition on CYP1A2-mediated phenacetin *O*-deethylation, CYP2A6-mediated coumarin 7-hydroxylation, CYP2C8-mediated amodiaquine *N*-deethylation, CYP2C9-mediated diclofenac 4-hydroxylation, CYP2C19-mediated [*S*]-mephenytoin 4′-hydroxylation, and CYP3A-mediated midazolam 1′-hydroxylation. IC_50_ values of corydaline and palmatine, the marker compounds of DA-9701 for CYP2D6-catalyzed bufuralol 1′-hydroxylation activity were 23.8 [23] and 49.3 *μ*g/mL (our unpublished data), respectively.

Although corydaline and palmatine inhibited CYP2D6 isozyme, they are not the major constituents causing the inhibition of CYP2D6 activity, because their contents in DA-9701 (corydaline, 0.85 *μ*g/mg and palmatine, 0.13 *μ*g/mg DA-9701) and Corydalis tuber extract (corydaline, 1.12 *μ*g/mg and palmatine, 0.18 *μ*g/mg Corydalis tuber extract) are very low. Therefore, other constituents may contribute to the inhibition of CYP2D6 activity by DA-9701 and Corydalis tuber extract. Further studies are needed to explore how the constituents of those compounds contribute to the inhibition of human CYP2D6 isozyme. The inhibitory potencies of DA-9701, Corydalis tuber extract, and Pharbitidis semen extract were not significantly affected after a 30-min period of preincubation with human liver microsomes in the presence of NADPH (Table 1), suggesting that a mechanism-based inhibitory component was not present in DA-9701, Corydalis tuber extract, and Pharbitidis semen.

Volume per dose index (VDI) is defined as the volume in which one dose would be diluted to obtain the corresponding IC_50_ concentration, as described by Strandell et al. [24], to determine the potential for *in vivo* inhibition of herbal preparations. If VDI value for a herbal preparation approaches 4 L, corresponding to human blood volume, the potential enzyme interaction of herbal preparation with pharmaceuticals should be further investigated [24]. Since a recommended human dose for DA-9701 is 30 mg, VDI value of DA-9701 for inhibition of CYP2D6 activity was 1.16 L/dose, suggesting that DA-9701 may not be a potent CYP2D6 inhibitor.

**Figure 1:**
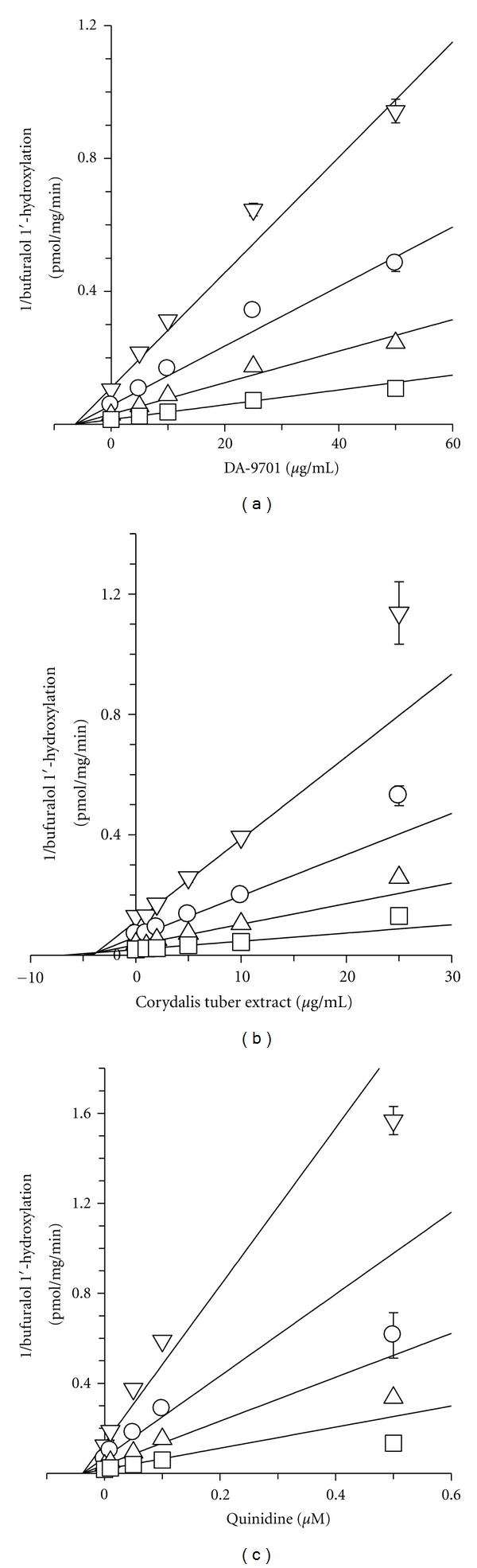
Representative Dixon plots for inhibitory effects of (a) DA-9701, (b) Corydalis Tuber extract, and (c) quinidine on CYP2D6-catalyzed bufuralol 1′-hydroxylation in pooled human liver microsomes, H161. Each symbol represents the bufuralol concentration: 0.5 *μ*M (*∇*), 1.0 *μ*M (◯), 2.0 *μ*M (∆), and 5.0 *μ*M (□). Each data point represents the mean of triplicate experiments.

In an inhibition study, the apparent *K*_i_ value is a better parameter for defining the interaction of the inhibitor with a particular enzyme. *K*_i_ values and inhibition types (competitive, noncompetitive, uncompetitive, or mixed) for DA-9701 and Corydalis tuber extract were initially estimated by graphical methods such as Lineweaver-Burk plot and Dixon plot, but they were ultimately determined by nonlinear least-square regression analysis for the best enzyme inhibition model using Enzyme kinetics software. DA-9701 showed noncompetitive inhibition for CYP2D6-catalyzed bufuralol 1′-hydroxylation, with a *K*_*i*_ value of 6.3 *μ*g/mL (Table 2, Figure 1). Corydalis tuber extract competitively inhibited CYP2D6-catalyzed bufuralol 1′-hydroxylation, with a *K*_i_ value of 3.7 *μ*g/mL (Table 2, Figure 1). The inhibitory potential of corydaline on CYP2D6 activity (*K*_i_ value of 10.1 *μ*g/mL (27.3 *μ*M)) [23] was not found to be strong, as compared with the inhibition produced by quinidine [*K*_i_ value of 12 ng/mL (0.038 *μ*M)], a selective inhibitor of CYP2D6 [25, 26].

We also evaluated the inhibitory potential of DA-9701, Corydalis tuber extract, and Pharbitidis semen extract on four major UGT isoform activities. DA-9701 and Corydalis tuber extract inhibited UGT1A1-mediated etoposide glucuronidation, with IC_50_ values of 188 and 290 *μ*g/mL, respectively. UGT1A4-mediated trifluoperazine *N*-glucuronidation, UGT1A9-mediated propofol glucuronidation, and UGT2B7-mediated azidothymidine glucuronidation were not inhibited by treatment with DA-9701 and Corydalis tuber extract (Table 3). Pharbitidis semen extract showed no inhibition of UGT1A1, UGT1A4, UGT1A9, and UGT2B7 activities in human liver microsomes (Table 3). VDI value of DA-9701 for inhibition of UGT1A1 activity was 0.16 L/dose, suggesting that DA-9701 may not inhibit *in vivo* UGT1A1 activity. This was supported by the comparison with IC_50_ values of UGT1A1 inhibitors, such as ritonavir (IC_50_ = 1.7 *μ*M) and ketoconazole (IC_50_ = 7.3 *μ*M) [27].

CYP2D6 catalyzes oxidation of a wide range of substrates, including desipramine, dextromethorphan, haloperidol, *S*-metoprolol, nortryptyline, paroxetine, and tamoxifen (http://medicine.iupui.edu/clinpharm/ddis/). Compared to quinidine, a known inhibitor of CYP2D6-catalyzed bufuralol 1′-hydroxylation [25, 26, 28], DA-9701 is a weak inhibitor (*K*_i_, 12 ng/mL versus 6.3 *μ*g/mL). On the basis of an IC_50_ value and VDI value of 1.16 L for a 30 mg dose, DA-9701 will be expected to have weak, if any, inhibition of CYP2D6-catalyzed metabolism *in vivo*. However, no pharmacokinetic data on DA-9701 are available. False prediction of *in vivo* drug-drug interactions from *in vitro* data may be occurred as a result of mechanism-based inhibition, plasma protein binding of inhibitors, production of inhibitory metabolites, and/or hepatic uptake. Therefore, the results need to be confirmed in clinical studies.

## 4. Conclusions

In conclusion, the effects of DA-9701 and its component herbs, Corydalis tuber extract and Pharbitidis semen extract, on seven CYPs and four UGTs were evaluated across a wide range of substrates using human liver microsomes *in vitro*. Pharbitidis semen extract showed no inhibition of seven CYPs and four UGTs in human liver microsomes. CYP2D6 activity was moderately inhibited by DA-9701 and Corydalis tuber extract during incubation with NADPH in human liver microsomes. On the basis of a *K*_*i*_ value and VDI value, DA-9701 will be expected to have weak, if any, inhibition of CYP2D6-catalyzed metabolism *in vivo*. Those results suggest that high uptake of botanical drug DA-9701 or Corydalis tuber extract may cause an interaction with drugs metabolized by CYP2D6 in some individuals. It is important to note, however, that the inhibition of CYP activities *in vitro* does not necessarily translate into drug interactions in clinical situations. Clinical trials to evaluate the inhibitory effects of DA-9701 on CYP2D6 remain to be conducted.

## Figures and Tables

**Table 1:** Effects of DA-9701, Corydalis tuber extract (CT Ex), and Pharbitidis semen extract (PS Ex) on CYP metabolic activity in pooled human liver microsomes, H161 using cocktail substrate assay.

CYP activity	CYP	IC_50_, *μ*g/mL (VDI**, L/dose)					
		No preincubation			With preincubation*		
		DA-9701	CT Ex	PS Ex	DA-9701	CT Ex	PS Ex
Phenacetin *O*-deethylation	1A2	N.I.***	N.I.	N.I.	N.I.	N.I.	N.I.
Coumarin 7-hydroxylation	2A6	N.I.	N.I.	N.I.	N.I.	N.I.	N.I.
Amodiaquine *N*-deethylation	2C8	N.I.	N.I.	N.I.	N.I.	N.I.	N.I.
Diclofenac 4-hydroxylation	2C9	N.I.	N.I.	N.I.	N.I.	N.I.	N.I.
*S*-mephenytoin 4′-hydroxylation	2C19	N.I.	145.9	N.I.	167.6 (0.18)**	130.3	N.I.
Bufuralol 1′-hydroxylation	2D6	25.9 (1.16)**	15.8	N.I.	34.3 (0.88)**	16.7	N.I.
Midazolam 1′-hydroxylation	3A4	N.I.	N.I.	N.I.	N.I.	N.I.	N.I.

*DA-9701, Corydalis tuber extract (CT Ex), and Pharbitidis semen extract (PS Ex) were preincubated for 30 min in the presence of NADPH before the addition of the substrate. **VDI: volume per dose index, ***N.I.: no inhibition at 200 *μ*g/mL of DA-9701. Cocktail substrate concentrations used for the assessment of IC_50_ were as follows: 50 *μ*M phenacetin, 2.5 *μ*M coumarin, 2.5 *μ*M amodiaquine, 10 *μ*M diclofenac, 100 *μ*M (*S*)-mephenytoin, 5.0 *μ*M bufuralol, and 2.5 *μ*M midazolam. The data represent the average of three determinations.

**Table 2:** *K*_i_ values for the inhibition of CYP2D6-catalyzed bufuralol 1′-hydroxylation activity by DA-9701, Corydalis tuber extract, and quinidine in pooled human liver microsomes, H161.

Substances	*K*_i_	Inhibition mode
DA-9701	6.3 *μ*g/mL	Noncompetitive
Corydalis tuber extract	3.7 *μ*g/mL	Competitive
Quinidine	0.038 *μ*M	Noncompetitive

**Table 3:** Effects of DA-9701, Corydalis tuber extract, and Pharbitidis semen extract on UGT metabolic activity in pooled human liver microsomes, H161.

UGT	Marker enzyme	IC_50_ (*μ*g/mL)		
		DA-9701	Corydalis tuber extract	Pharbitidis semen extract
UGT1A1	Etoposide glucuronidation	188	290	No inhibition*
UGT1A4	Trifluoperazine *N*-glucuronidation	No inhibition	No inhibition	No inhibition
UGT1A9	Propofol glucuronidation	No inhibition	No inhibition	No inhibition
UGT2B7	Azidothymidine glucuronidation	No inhibition	No inhibition	No inhibition

*There was no inhibition at 200 *μ*g/mL of DA-9701, Corydalis tuber extract, and Pharbitidis semen extract. The data represent the average of duplicate analysis.
